# T cell subsets and immunoglobulin G levels are associated with the infection status of systemic lupus erythematosus patients

**DOI:** 10.1590/1414-431X20154547

**Published:** 2017-12-11

**Authors:** Lifen Wu, Xinru Wang, Fenghua Chen, Xing Lv, Wenwen Sun, Ying Guo, Hou Hou, Haiyan Ji, Wei Wei, Lu Gong

**Affiliations:** 1 Department of Ultrasonography, Tianjin Medical University General Hospital, Tianjin, China; 2 Department of Medical Clinical Laboratory, The General Hospital of People's Liberation Army Rocket Force, Beijing, China; 3 Department of Obstetrics and Gynecology, Reproductive Medical Centre, Peking University Third Hospital, Beijing, China; 4 Department of Rheumatology, Tianjin Medical University General Hospital, Tianjin, China

**Keywords:** Systemic lupus erythematosus, Infection, T cell subsets, Immunoglobulin G

## Abstract

Systemic lupus erythematosus (SLE) is a chronic, autoimmune disorder that affects nearly all organs and tissues. As knowledge about the mechanism of SLE has increased, some immunosuppressive agents have become routinely used in clinical care, and infections have become one of the direct causes of mortality in SLE patients. To identify the risk factors indicative of infection in SLE patients, a case control study of our hospital's medical records between 2011 and 2013 was performed. We reviewed the records of 117 SLE patients with infection and 61 SLE patients without infection. Changes in the levels of T cell subsets, immunoglobulin G (IgG), complement C3, complement C4, globulin, and anti-double-stranded DNA (anti-ds-DNA) were detected. CD4+ and CD4+/CD8+ T cell levels were significantly lower and CD8+ T cell levels were significantly greater in SLE patients with infection than in SLE patients without infection. Additionally, the concentrations of IgG in SLE patients with infection were significantly lower than those in SLE patients without infection. However, complement C3, complement C4, globulin, and anti-ds-DNA levels were not significantly different in SLE patients with and without infection. Therefore, clinical testing for T cell subsets and IgG is potentially useful for identifying the presence of infection in SLE patients and for distinguishing a lupus flare from an acute infection.

## Introduction

Systemic lupus erythematosus (SLE) is a chronic, autoimmune disorder that affects nearly all organs and tissues; this disease is severe and life threatening (1). SLE is characterized by an elevated presence of autoantibodies, the formation of immune complexes, and the involvement and damage of multiple organ systems. Many genetic, hormonal, and environmental factors are known to affect the development and nature of this disease. SLE affects females at a far greater rate than males; however, SLE in males is often more severe than it is in females. Gender disparities have also been reported in SLE clinical manifestations, and in serological and hematological indices (2,3).

The death of patients with SLE may be caused by lupus activity when vital organs or systems are involved, by treatment complications (particularly infections), or by long-term sequelae, such as cardiovascular disease. Studies have reported that lupus nephritis (LN) is the current leading cause of morbidity and mortality in SLE patients; LN develops in 50 to 75% of Asian SLE patients (4). Similarly, Contreras et al. found increased risks of the doubling of creatinine levels, progression to end-stage renal disease, and death in African Americans and Hispanics compared to Caucasians (5). A study conducted in the United Kingdom also confirmed ethnic disparities in the incidence of renal failure; 62% of the renal failure patients in that study were of African descent (6). However, as knowledge of the SLE mechanism progressed, new therapeutic targets were identified. New drugs that have allowed SLE patients to live longer are routinely used clinically. The mortality rate of LN patients has been greatly reduced. Furthermore, the prognosis of SLE patients has greatly improved, and the 10-year survival rate of SLE patients has significantly increased. However, the new clinical drugs have increased the incidence of infection in SLE patients. Infection is responsible for approximately 25% of all SLE patient deaths; thus, it is the second leading cause of SLE mortality after cardiovascular death (7–10).

As SLE patients are a high-risk population, it is important to identify and treat chronic infections, such as tuberculosis, hepatitis B, and human immunodeficiency virus (HIV), prior to initiating immunosuppressive drugs to prevent the reactivation of chronic infections. It is important to distinguish between a lupus flare and an acute infection. The judicious use of corticosteroids and cytotoxic drugs is critical in limiting infectious complications. Lertchaisataporn et al. reported that leukopenia in SLE patients at any time is not a risk factor for severe infection; the authors found that cyclophosphamide was an important predictor of severe infection in SLE (11). In contrast, Merayo-Chalico et al. (12) suggested that lymphopenia, prednisone treatment, and low levels of complement C3 were independent risk factors for the development of a set of diverse and severe infections (not only opportunistic infections) in SLE patients. The present study evaluated changes in the frequency of different subsets of T cells, and the levels of serum immunoglobulin G (IgG), complement C3, complement C4, anti-ds-DNA, and globulin in SLE patients with and without infection to determine whether they were associated with the infection in SLE patients.

## Material and Methods

### Patients

We enrolled 175 female and 3 male SLE patients from the Tianjin Medical University General Hospital medical records between 2011 and 2013, with ages between 18 and 71 years (mean 36.73±13.64). All patients fulfilled the updated American College of Rheumatology classification criteria for SLE (13) and the disease severity of individual patients was evaluated, according to SLE disease activity index (SLEDAI). The inclusion criteria for the study were: SLE patients >18 years, regardless of gender, duration and severity of disease, therapeutic experience and the presence or absence of infection. The exclusion criteria were: 1) SLE patients with concomitant malignant diseases; 2) SLE patients with HIV infection; 3) SLE patients with a history of other autoimmune diseases; 4) Pregnant females. The study was approved by the Institutional Review Board of our hospital.

### Definition of infection

The patients were diagnosed with infections when bacteria, fungi or viruses could be isolated from cultures of blood or cerebrospinal fluid in a sterile area or when specific antibodies were identified using polymerase chain reaction (PCR). For patients with negative culture results, infection was diagnosed through typical symptoms (e.g., fever, cough, new onset of purulent sputum, urgency, dysuria, and purulent drainage at affected site), signs (e.g., wheezing, rales, rhonchi, and abdominal or suprapubic tenderness), radiological evidence (e.g., evidence of an abscess on ultrasound, computed tomography, or magnetic resonance imaging), laboratory evaluations (e.g., leukocytosis and left shift, positive urine dipstick for leukocyte esterase and/or nitrate or pyuria), and other evidence of bacterial infection, such as an abscess, observed during surgery or histopathological examination. A positive response to the standard antibacterial therapy was also used to support the diagnosis of bacterial infection. Infections were categorized as those requiring intravenous antimicrobial therapy, such as pneumonia, urinary tract infection, skin and soft tissue infection, and sepsis, and those caused by opportunistic pathogens, such as Mycobacterium species, herpes zoster, cytomegalovirus (CMV), and *Pneumocystis jiroveci*. Of the 178 patients enrolled in this study, 117 patients had infections, and 61 did not.

### AESKULISA ds-DNA-G ELISA test

AESKULISA ds-DNA-G is a solid-phase enzyme immunoassay that uses human recombinant double-stranded DNA (ds-DNA) to quantitatively detect IgG antibodies against ds-DNA in human serum (AESKULISA, dsDNA G, Aesku Diagnostics, Germany) (14). Serum samples (diluted 1:100) were incubated in a microplate coated with a specific antigen. If specific antibodies were present in a patients' blood, they bound to the antigen; subsequently, the unbound fraction was washed away. The microplate was then incubated with anti-human immunoglobulin conjugated to horseradish peroxidase (conjugate), which reacted with the antigen-antibody complex of the samples in the microplate; any unbound conjugate was washed off in the following step. The addition of TMB-substrate generated an enzymatic colorimetric (blue) reaction, which was stopped by adding diluted acid. The rate of color formation from the chromogen was a function of the amount of conjugate bound to the antigen-antibody complex, and this rate was proportional to the initial concentration of the respective antibodies in the patient sample. The reference value of anti-dsDNA for healthy subjects was <2 IU/mL.

### Nephelometry

Nephelometric measurements for the quantification of the complement C3 and complement C4 levels and IgG levels were performed using a Siemens-BN-II nephelometer (Beckman Coulter, USA). The intensity of dispersed light was proportional to the concentrations of complement C3, complement C4, and IgG. The reference values were 79–152 mg/dL for complement C3, 16–38 mg/dL for complement C4, and 751–1560 mg/dL for IgG (15).

### Flow cytometry

Lymphocyte subset analyses for all patients were performed using a BD-FACS-CANTO II flow cytometer (Becton Dickinson, USA). The principle of flow cytometry is based on laser light dispersion, sensitization, and cellular detection with fluorochrome molecules. Blood samples from all subjects were stained and analyzed within 6 h of collection. A whole-blood lysing method was used, and the remaining leukocytes were then stained using monoclonal antibodies that had been directly conjugated to fluorescein (FITC), phycoerythrin (PE), and peridinin-chlorophyll-protein complex (PerCP). Lymphocyte subset analyses for all patients were performed by direct three-color immunofluorescence and flow cytometry. The following mouse IgG1 monoclonal antibodies with appropriate isotype controls were used: anti-human CD3 (PerCP), anti-human CD4 (FITC), and anti-human CD8 (PE). Diva software was used to obtain the percentages of the lymphocyte subsets (16). The reference values of the percentages of CD3+, CD4+ and CD8+ T cells as well as the ratios of CD4/CD8 T cells were 61–85, 34–70, 25–54, and 0.68–2.47%, respectively.

### Statistical analysis

Data from the patients with infection and the controls were compared using the independent-sample *t*-test and the chi-square test for quantitative variables. The Shapiro-Wilk test was used to assess normality. As most of the data were not normally distributed, the Mann-Whitney U test was used to examine differences in the non-parametric data between the patients with infections and the controls. The hypothesis was examined using two-tailed tests. P values ≤0.05 were considered statistically significant. All analyses were performed using Empower (R; www.empowerstats.com, X&Y Solutions, Inc., USA) and R (http://www.R-project.org).

## Results

### Patient characteristics

Among the 178 patients recruited in this study, 117 had infection, and 61 had no infection. The demographic and clinical characteristics, including gender, age, and SLE duration, did not differ between the SLE patients with and without infection. Among the patients with infection, 60 patients had pneumonia, 5 had urinary tract infection, 20 had skin and soft tissue infections, 10 had multiple-site infections (pneumonia and urinary tract infection or sepsis), 4 had sepsis, and 18 had viral infections (including herpes zoster, CMV, human papillomavirus, and parvovirus B 19; Table 1). The types of therapy that the SLE patients with and without infection underwent are shown in Table 1.

**Table 1.** Demographic data and clinical characteristics of SLE patients (n=178).

Clinical parameters	Infected (n=117)	Non-infected (n=61)	P value
Age (years)	38.28±14.40	34.04±11.86	0.19
Gender, n (%)			
Male	1 (0.9%)	2 (3.3%)	0.27
Female	116 (99.1%)	59 (96.7%)	
Duration of SLE (year)	3.52±4.22	3.49±6.48	0.98
SLEDAI	11.77±8.9	13.47±9.5	0.50
Pneumonia	60	–	–
Urinary tract infection	5	–	–
Skin and soft tissue infection	20	–	–
Multiple site infection	10	–	–
Sepsis	4	–	–
Virus	18	–	–
Glucocorticoid	117 (100%)	5 (8.2%)	0.22
Cyclophosphamide	20 (17.09)	2 (3.28%)	0.08
Cyclosporine A	2 (1.71%)	4 (6.56%)	0.89
Mycophenolate mofetil	3 (2.56%)	59 (96.72%)	0.18
Hydroxychloroquine	57 (48.72%)	35 (57.38%)	0.27
Leflunomide	13 (11.11%)	3 (4.92%)	0.27

Data are reported as means±SD or number and percentage. SLE: systemic lupus erythematosus; SLEDAI: systemic lupus erythematosus disease activity index (Mann-Whitney U or *t*-test).

### T cell subsets in SLE patients with and without infection

The types of infections found in SLE patients are similar to those found in the general population and are caused by the same pathogens (Gram-positive and Gram-negative bacteria and viruses). SLE patients may also develop opportunistic infections, particularly when treated with immunosuppressive agents.

**Figure 1.**
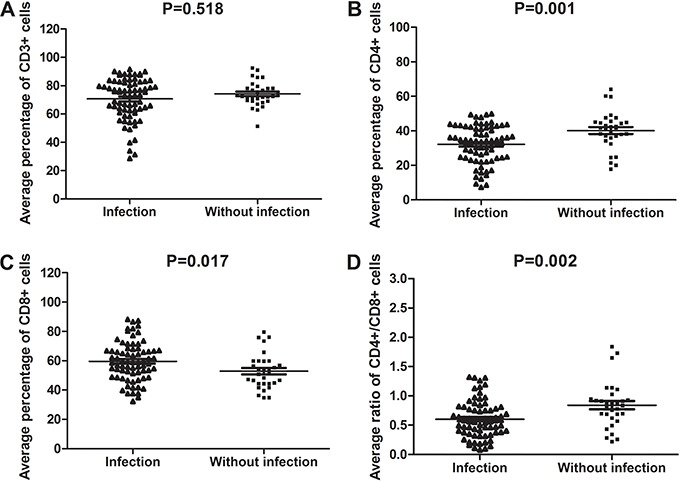
T cell subset levels in 73 systemic lupus erythematosus (SLE) patients with infection and 31 SLE patients without infection. Flow cytometry was used to test T cell subsets. *A*, percentage of CD3+ T cells. *B*, percentage of CD4+ T cells. *C*, percentage of CD8+ T cells. *D*, CD4+/CD8+ T cells ratio (Mann-Whitney U or t-test).

Based on those findings, we investigated alterations in T cell subsets in SLE patients. T cell subset data were collected for 73 SLE patients with infection and for 31 SLE patients without infection. The average level of CD3+ cells in SLE patients with infection was not significantly different from that in SLE patients without infection (mean rank, 51.3 *vs* 55.4, U=1040.5, P=0.518; Figure 1A). However, the average percentage of CD4+ T cells in SLE patients with infection was significantly lower than that in SLE patients without infection (32.1 *vs* 40.2%, P=0.001; Figure 1B). In contrast, the average CD8+ T cell percentage was significantly greater in SLE patients with infection compared to those without infection (59.6 *vs* 52.8%, P=0.017; Figure 1C). The average CD4+/CD8+ ratio was significantly lower in SLE patients with infection than in those without infection (mean rank, 46.48 *vs* 66.68, U=692.0, P=0.002; Figure 1D).

### IgG, anti-ds-DNA, and globulin in SLE patients with and without infection

**Figure 2.**
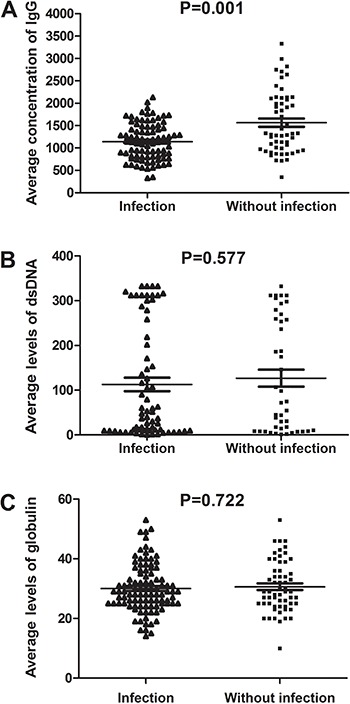
The concentrations of IgG, anti-ds-DNA, and globulin in systemic lupus erythematosus (SLE) patients with or without infection. *A*, data were obtained from 86 SLE patients with infection and 55 SLE patients without infection. *B*, 68 SLE patients with infection and 44 SLE patients without infection. *C*, SLE patients with infection (n=99) and SLE patients without infection (n=59) (Mann-Whitney U or t-test).

For the IgG level analysis, data were obtained from 86 SLE patients with infection and from 55 SLE patients without infection. The concentrations of IgG in SLE patients with infection were significantly lower than in the patients without infection (mean rank, 61.43 *vs* 85.96, U=1542.0, P=0.001; Figure 2A). For the anti-ds-DNA level analysis, samples were obtained from 68 SLE patients with infection and 44 SLE patients without infection. The concentrations of anti-ds-DNA exhibited only a slight difference between SLE patients with infection and those without infection (mean rank, 55.12 *vs* 58.62, U=1402.5, P=0.577; Figure 2B). We also analyzed the globulin levels in 99 SLE patients with infection and 59 SLE patients without infection. Little disparity in the levels of globulin was found between the SLE patients with and without infection (mean rank, 78.5 *vs* 81.18, P=0.722; Figure 2C).

### Complement C3 and complement C4 in SLE patients with and without infection

**Figure 3.**
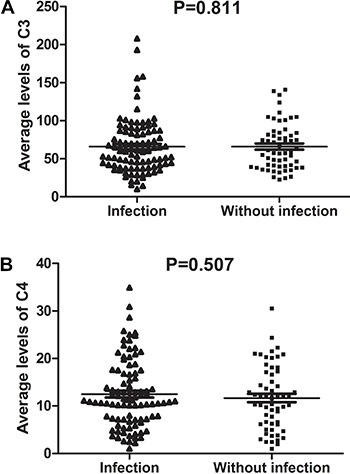
Complement C3 (*A*) and C4 (*B*) levels determined by nephelometry in the serum specimens of systemic lupus erythematosus patients with or without infection (Mann-Whitney U or t-test).

We analyzed the serum complement C3 and complement C4 levels in 97 SLE patients with infection and in 59 SLE patients without infection and found that the levels of complement C3 in SLE patients with infection were similar to those in patients without infection (mean rank, 77.82 *vs* 79.61, U=2796.0, P=0.811; Figure 3A). The levels of complement C4 in SLE patients with infection were also similar to those in patients without infection (mean rank, 79.88 vs 74.95, U=3012.0, P=0.507; Figure 3B).

## Discussion

In this study, we investigated the hospitalized SLE patients in our center and explored the characteristics of and different factors among SLE patients with and without infection. SLE is a typical autoimmune disease involving multiple systems and organs. The use of biologic immunosuppressants for the treatment of SLE has been increasing, but it may increase the infection risk for SLE patients (17). Infection is one of the leading causes of morbidity and mortality in SLE patients. Often, infections result in hospitalization and/or death among these patients (18,19). The prevalence of life-threatening infections appears to be greatest within the first 5 years of disease onset (20). It is challenging to determine whether a fever is due to a superimposed infection or to the SLE disease activity itself. Our work aimed to determine the correlations between certain indicators of SLE and infection and to analyze the clinical significance of these indicators in SLE patients with infection. Lertnawapan et al. (21) reported that *Pneumocystis Carinii* pneumonia (PCP)-infected SLE patients had lower lymphocyte and CD4+ counts than patients without PCP infection. Dias et al. (22) showed that neutropenia was associated with an increased risk of infection during a 1-year follow-up study. Accumulating evidence, however, indicates that aberrant T cell subsets also play a unique role in the progression of infection in transplantation (23). T cell subset comparisons between SLE patients with and without infection have not yet been reported. In our study, we found that the CD4+ cell number and the CD4+/CD8+ ratio were lower in SLE patients with infection than in those without infection. Wang et al. (24) have shown that CD4+, CD8+ T cells and CD4+/CD8+ ratio from SLE patients are significantly decreased compared with normal controls. CD4+ T cells have been reported to play a central role in the control of autoimmunity, immune homeostasis, and immune responses to pathogens and tumor antigens (25). Our results are consistent with the roles of CD4+ T cells in mediating the immune response and in eliminating viral, bacterial, fungal, and parasitic infections and malignant cells.

Previous reports have indicated that IgG and anti-ds-DNA levels have a strong association with disease activity throughout the course of SLE (26,27). We also found that IgG was lower in SLE patients with infection than in those without infection. IgG was lower in normal control than in SLE. But the levels of IgG between SLE patients with infection and normal control are unclear. It is well known that IgG is one of the most abundant antibody isotypes found in the circulation and that it can control infections in body tissues. Polilli et al. (28) reported that the intravenous infusion of standard human immunoglobulin significantly increased CMV IgG titers and avidity indexes in pregnant women; these results support the use of immunoglobulin for the passive transfer of maternal CMV humoral immunity to fetuses. Our results are consistent with the known functions of IgG; thus, IgG levels can be used as indicators of the use of immunosuppressive agents. Typically, globulin levels are greater in SLE patients than in the general population. Two additional SLE diagnostic biomarkers of disease activity are the serum complement C3 and complement C4 levels. Therefore, we analyzed globulin, complement C3 and complement C4 levels in SLE patients with and without infection. The complement system is involved in both the development of SLE and in mediating the pathological effects of the autoantibodies (29). It has been reported that a low level of complement C3 is an independent risk factor for the development of severe infection in SLE patients (10). According to our data, there was little variation in the complement C3 levels between SLE patients with and without infection. Our results may have been affected by the inclusion criteria used for the patients with infection. A historical comparison of SLE patients with and without infection demonstrated improved patient survival in the absence of concomitant infection. All SLE patients with fever should be tested for these markers. If the percentage of CD4+ T cells, CD4+/CD8+ ratio, and IgG concentration are low, the physician should consider the possibility of infection. We believe that the T cell subsets and IgG concentration correlate with the infection status of SLE patients.

The present study had some limitations. First, it was retrospective in nature, and only some of the identified patients with or without infection were subjected to the measurement of T cell subsets, anti-dsDNA, complement C3, complement C4, IgG, and globulin. Second, in the hospitalized patients, the T cell subset was monitored primarily when the presence of infection was considered, and the absence of T cell subset testing among patients without infection likely introduced bias. Furthermore, all patients were from one medical center.

In conclusion, the CD4+ T cell number and the CD4+/CD8+ ratio were lower in SLE patients with infection compared with patients without infection. The IgG concentration was significantly lower in SLE patients with infection than in patients without infection. However, the concentrations of anti-ds-DNA, complement C3, complement C4, and globulin did not significantly differ between SLE patients with and without infection.
